# Assessment of math anxiety as a potential tool to identify students at risk of poor acquisition of new math skills: longitudinal study of grade 9 Italian students

**DOI:** 10.3389/fpsyg.2023.1185677

**Published:** 2023-07-14

**Authors:** Massimo Piccirilli, Gianni Alberto Lanfaloni, Livia Buratta, Beatrice Ciotti, Alessandro Lepri, Cristina Azzarelli, Silvia Ilicini, Patrizia D’Alessandro, Sandro Elisei

**Affiliations:** ^1^Department of Medicine, University of Perugia, Perugia, Italy; ^2^Serafico Institute, Assisi, Perugia, Italy; ^3^Department of Philosophy, Social Sciences and Education, University of Perugia, Perugia, Italy

**Keywords:** math anxiety, AMAS, math achievement, adolescence, educational psychology, mathematics performance, secondary school students

## Abstract

**Introduction:**

Numerous international educational institutions have sounded the alarm about the gradual increase in the number of students failing to achieve a sufficient level of proficiency in mathematical abilities. Thus, the growing interest in identifying possible solutions and factors interfering with learning seems justified. In recent years, special attention has accrued to the possible role played by emotional factors.

**Methods:**

In the present investigation, students in the first grade of a technical vocational secondary school are followed to assess the influence of math anxiety (MA) on the development of skill acquisition in calculus. A math skills assessment test is administered on two occasions, at the beginning and end of the school year.

**Results:**

Results highlighted that the score on the anxiety scale, administered at the beginning of the year, negatively correlated with the score obtained on the mathematics test, administered at the end of the school year: the higher the level of anxiety, the worse the performance. Furthermore, the score obtained in the second administration makes it possible to divide the students tested into two groups: students who improved their performance and students who did not benefit at all from repeating the test. In these two groups, an analysis of the relationships between the outcome of the end-of-year mathematics test and the level of MA at the beginning of the year showed that MA correlates negatively with performance only in students who will fail to acquire new expertise in mathematics over the course of the school year.

**Discussion:**

The results suggest that MA may interfere with the smooth development of math skills. Assessing the level of MA at the beginning of the school year could prove to be a useful tool in identifying which and how many students are at risk of failing to achieve the skills expected from the usual course of instruction. A consideration of anxiety as one of the variables at play in the genesis of learning difficulties may prompt educators to modify teaching methodology and strategies by increasing focus on the impact of the emotional dimension on learning.

## Introduction

The invention of numbers represents a milestone in human history that ultimately led, through progressive developments that took place over the past 4,000 years, to the number system currently in use (Boyer and Merzbach, 2010). At an individual level, the acquisition of mathematical skills turns out to be a highly complex process (Butterworth, 1999). Indeed, a newborn infant possesses the ability to distinguish between numbers with different values (e.g., one, two, and many), but genetic heritage is not sufficient to allow the manipulation of numbers (Dehaene, 1996). Achieving this manipulation requires a long period of learning, which in our society is mediated by teaching through compulsory schooling (Dehaene, 2021). That is why mathematics is one of the main domains of the school curriculum. A substantial proportion of subjects, however, struggle when confronted with contexts in which it is necessary to show some knowledge of mathematics (Fennema and Sherman, 1976). Difficulties in learning mathematics may indicate a clinical issue—a specific disorder called dyscalculia. In dyscalculia, the origin of the deficit is cognitive (Lucangeli, 1999; Passolunghi, 2011). However, even those who do not suffer from a cognitive disorder may demonstrate a negative attitude when faced with the need to study mathematics. In fact, the number of students who at the end of their schooling show that they have not achieved the minimum skills needed for everyday life appears to be constantly rising (Invalsiopen, 2022; Save the Children, 2022).

Numerous attempts have been made to understand the causes of these widespread difficulties in mathematics and, in recent years, great prominence has been given to math anxiety (MA), a concept introduced by Dreger and Aiken (1957) and later defined by Richardson and Suinn (1972) as “feelings of tension and anxiety that interfere with the manipulation of numbers and the solving of mathematical problems in a wide variety of ordinary life and academic situations.” This negative reaction may manifest itself in various ways (Hembree, 1990): at an emotional level (as feelings of discomfort, apprehension, aversion, worry, frustration, or fear), at a physical level (as malaise, tachycardia, muscle tension, shortness of breath or sweating, and neuro vegetative reactivity) or at a behavioral level (as refusal to go to school or avoidance of homework and study). MA’s severity may be such that it can result in a true phobia, namely, an unreasonable fear when faced with real or even imaginary number-related situations (Gough, 1954).

Implicit in the definition of MA is that it is a specific form of anxiety, distinct from state anxiety and trait anxiety as well as the anxiety that may occur when one has to perform activities other than mathematics, such as public speaking in the case of social phobia. The subsequent literature, with some exceptions, seems to have confirmed the specificity of MA (Hembree, 1990; Devine et al., 2012; Carey et al., 2017; Choe et al., 2019; Pizzie and Kraemer, 2019), and its theoretical structure as an autonomous entity has been further corroborated by the identification of its neurobiological basis (Lyons and Beilock, 2012; Young et al., 2012; Artemenko et al., 2015; Supekar et al., 2015; Suárez-Pellicioni et al., 2016).

Also implicit in the definition is that MA may adversely affect the ability to solve mathematical problems. Indeed, it is sufficiently well documented that subjects with high levels of MA tend to have poor performance in mathematics tasks although they are able to learn other school subjects in an adequate manner (Hembree, 1990; Ma, 1999; Ashcraft and Krause, 2007; Maloney et al., 2011; Barroso et al., 2021; Dowker and Sheridan, 2022).

In our previous study of first-year technical vocational secondary school students, we examined the relationships between performance on a standardized test to assess different mathematics skills and anxiety, assessed as trait anxiety, state anxiety, and math-specific anxiety (Buratta et al., 2019). The math test is aimed at evaluating the functional architecture of numerical processing mechanisms, according to the model of numerical cognition proposed by McCloskey et al. (1985): various subtests present problems of increasing complexity useful for measuring the level of ability achieved by the student on the basis of the school grade. Data, derived by simultaneous multivariate linear regression analysis exploring the role of the single anxiety scales (maths, trait, and state) on the mathematics performance scores, highlighted that MA was the only variable that was significantly linked to the arithmetical performance. Specifically, MA was not linked with basic math performance but with task assessing more advanced competence. On the contrary, math performance was not affected by the presence of trait or state anxiety.

In order to better understand this relevant issue, we have planned to reevaluate the same students at the end of their school year. To facilitate the analysis of the effects of anxiety on performance, we preferred to keep the difficulty of the mathematical test unchanged. As a matter of fact, having students face a more complex task in the second test than in the first would have made it difficult to differentiate the effects of anxiety from the consequences of increasing the difficulty of the task (Geary et al., 2019). For this purpose, at the end of the school year, the same students examined in the first stage of this investigation performed the same test that they had done at the beginning of the school year. Theoretically, at the end of their school year, students, having carried out the formal teaching activities foreseen by the institutional curriculum, should have acquired new knowledge and skills in mathematics. Consequentially, by running the same task for the second time at the end of the school year, all students should show an improvement in their performance, i.e., they should obtain a higher score on the second administration of the test. Contrariwise, a lower score in the second evaluation than in the first would indicate that the student has not benefited from the teaching offered during the school year. In our hypothesis, the presence of MA at the beginning of the school year could negatively interfere with the mathematical performance tested at the end of the course of study, despite the specific teaching activity received during the school year. Furthermore, we hypothesize that a high level of MA may hinder the ability to acquire new math skills and that the link to MA could be different for students who did not benefit at all from repeating the test than for students who improved their performance.

## Subjects and method

### Subjects

73 Italian-speaking students (59 male and 14 female) attending the first grade of a technical vocational secondary school (corresponding to grade nine of English-speaking schools and of the Italian INVALSI evaluation system) participated in this phase of the survey. The mean age at the time of the beginning of the school year, when students first participated in our study, was 14 years and 7 months (SD = 4 months, range = 14–15.1 years). Diagnosis of dyscalculia or a specific learning disorder (as documented by the certification issued by the health authority to the educational institution to organize a personalized treatment in accordance with the Italian guidelines on specific learning disabilities) was an exclusion criterion. The participants and their parents were informed about the purposes of the research and gave signed informed consent. Participation in the study did not include any type of reward. The study was approved by CEAS, the Local Ethics Committee of the Umbria Region of Italy.

### Procedure

Students were tested on two separate occasions, approximately 6 months apart, once in January at the end of the first term (T0) and a second time in June at the end of the second term (T1). This period corresponds in Italian schools to the moments in which formal tests are carried out to measure the level of learning achieved by the students. All participants first filled in the three questionnaires that assessed, in order, trait, math, and state anxiety; later they completed the mathematical tests. They did all measures individually in class during school hours and were aware that the data obtained would remain anonymous and would not be shared with their teachers.

### Measures

#### Abbreviated math anxiety scale (AMAS)

AMAS is a questionnaire consisting of nine items scored from 1 to 5 (on a five-point Likert scale); the score can thus range from a minimum value of 9 to a maximum value of 45, which is indicative of the highest possible level of math anxiety (Hopko et al., 2003). The items assess two distinct subscales: “Learning Math Anxiety” (five items that explore anxiety related to math study, such as “Watching the teacher break down a complex problem on the blackboard”) and “Math Evaluation Anxiety” (four items that explore anxiety related to assessment situations, such as “Doing a written math examination/test”). In this study, we used the Italian version which was validated by Primi et al. (2014). When administered to secondary school students, the Italian adaptation of AMAS exhibits psychometric properties similar to those of the original test with respect to internal consistency (Cronbach’s alpha ≥0.80) and test–retest reliability (Cronbach’s alpha ≥0.81); furthermore, the two dimensions established in the original AMAS (Learning Math Anxiety and Math Evaluation Anxiety) were evident also in the Italian version as well as the invariance across genders. In addition, transcultural validity of math anxiety assessment with the AMAS has been documented in numerous studies (Vahedi and Farrokhi, 2011; Cipora et al., 2015) and has allowed collaborative studies between researchers of different languages, for example, Italian and English (Hill et al., 2016; Mammarella et al., 2018; Carey et al., 2019; Wang et al., 2020).

#### State and trait anxiety inventory (STAI-Y)

STAI-Y is a 40-item questionnaire scored from 1 to 4 (on a four-point Likert scale) and consists of two subscales of 20 items that assess state anxiety, which is anxiety related to a specific moment or event (such as might be represented by anxiety in relation to the test), and trait anxiety, which is a condition related mainly to personality characteristics (Spielberger, 1989). In the first phase of this investigation (Buratta et al., 2019), the use of STAI-Y allowed us to differentiate the role of MA from that of other forms of anxiety and to establish that the levels of trait anxiety and state anxiety do not correlate with mathematical performance and do not influence it negatively. The Italian version of the questionnaire, which was validated by Pedrabissi and Santinello (1989), was used in this study: the internal consistency values is 0.91 for the State anxiety scale and 0.85 for Trait anxiety scale; the test–retest reliability is 0.49 for state subscale and 0.82 for trait anxiety. Given the good psychometric properties and its availability in different languages that make it useful for cross-cultural investigations, the STAI-Y is the best-known and most widely used self-report questionnaire to assess anxiety in research and clinical practice.

#### Battery for the assessment of calculation ability (ABCA 14-16)

ABCA 14-16 is a battery of paper-pencil tests for the assessment of mathematical skills in 14- to 16-year-old subjects (Baccaglini-Frank et al., 2013). This battery of tests has been constructed with reference to the modular model of McCloskey et al. (1985) which hypothesizes that the mental representation of numerical knowledge is independent of other cognitive systems and is structured in three modules which are in turn functionally distinct. The different tests in the battery provide a specific profile that identifies which components are deficient within an individual student’s mathematical skills comparing the percentile scores to the cut-off criteria of the normative sample. The items assess different levels of mathematical proficiency by requiring the solution of tasks of different complexity (for example, “145.28–23.39 =” or “57.8 × 2.94 =”). In the present study, we considered subtests that in the first phase of our investigation were affected by the level of MA. These subtests consist of advanced math skills that investigate how students have stored combinations of numbers within the calculation system and whether they are able to access them automatically (for example, “2 + 3 × 4 =” or “100/10^2^ =”). Scoring, which is based on the number of correct answers, ranges from a minimum of zero to a maximum of 28, and high scores correspond to superior performance. The ABCA test is widely used in Italy to assess the achievement of the educational objectives set by the school curriculum.

## Statistical analysis

The Java Structural Program was used for data analysis (JASP Team, 2022, Version 0.16.3). Descriptive statistics in terms of mean and standard deviation were employed to describe the scores obtained in the AMAS questionnaire and ABCA 14-16 test. The relationship between MA levels at T0 and math performance at T1 was examined using the Pearson correlation coefficient.

To further investigate this relationship, we divided the group of students into more homogeneous subgroups. As regards the AMAS questionnaire, the students who obtained scores above the mean by one standard deviation constituted the subgroup with high levels of anxiety (HMA), while the students with scores below the mean by one standard deviation constituted the subgroup characterized by low levels of anxiety (LMA). Student t-test was used to compare the scores obtained by the two subgroups in mathematics performance.

As regards math performance at T1, the score obtained in the math test at T1 was used as an indicator of the student’s ability to make use of the study period between the two assessments to acquire better math competence; a higher score on the second than on the first assessment identified the subgroup of students who improved performance, showing that they have learned new calculus skills (subgroup with improved math performance, IMP), while other students constituted the subgroup whose performance did not improve, showing that they have failed to acquire new calculus skill (subgroup with worsened math performance, WMP). A paired t-test was utilized to compare the mathematical performance in the two assessments at T0 and T1 and a linear regression, based on the AMAS questionnaire score at T0 as an independent variable and the math performance score at T1 as a dependent variable, was used to evaluate the possible influence exerted by the MA on math performance of the two subgroups of students.

## Results

The AMAS questionnaire indicated an average anxiety level of 21.79 ± 6.08. Subscale “Learning Math” showed an average score of 8.45 (SD = 2.77) and subscale “Math Evaluation” an average score of 13.42 (SD = 4.15). There were 15 (20, 27%) students belonging to the HMA subgroup characterized by high levels of anxiety (M = 30.33, SD = 1.88), while 14 (19, 18%) belong to the LMA subgroup characterized by low levels of anxiety (M = 14, SD = 1.07). HMA students showed significantly (*p* = 0.001) worse math performance (M = 12.66, SD = 7.28) than LMA students (M = 20.71, SD = 3.73).

As regards the mathematical test performed at T1, the average score obtained was 16.37 (SD = 6.3). The correlation coefficient showed an inverse relationship between the MA and the score obtained in the math test (*r* = −0.386, *p* = 0.002): the higher the MA level recorded at the beginning of the school year, the lower the score on the math test at the end of the year. This relationship persisted even considering separately the two “Learning Math” (*r* = −0.388, *p* = 0.002) and “Math Evaluation” (*r* = −0.306, *p* = 0.02) components of the AMAS questionnaire.

**Figure 1:**
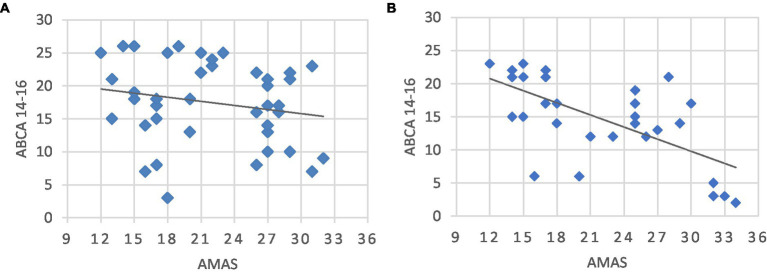
Relationship between perceived anxiety about mathematics and performance on the mathematics test. **(A)** Students whose performance improves over time (*r* = 0.194; *R*^2^ = 0.038; *p* = 0.22). **(B)** Students whose performance does not improve over time (*r* = −0.637; *R*^2^ = 0.406; *p* = 0.001). AMAS, score obtained at the beginning of the school year on the on the Math Anxiety Assessment Questionnaire; ABCA 14-16, score obtained at the end of the school year on the Italian battery for the assessment of calculation ability.

In the interpretation of this inverse correlation, it must be kept in mind that an increase in the score in the second administration of the mathematical test compared to the first can be interpreted as an index of the acquisition of new math skills resulting from the teaching received during the school year. Conversely, the deterioration of performance, i.e., the decrease in the score obtained when the test was administered for the second time, can be interpreted as the consequence of the inability to acquire better math skills. Actually, there were 42 (57.53%) belonging to the IMP subgroup characterized by an improvement in math performance from an average score of 12.4 ± 7.1 to 17.47 ± 6.18 (*t* = 8.93; *p* < 0.001), while the remaining 31 students (42.47%) belonged to the WMP subgroup characterized by a math performance deterioration from an average score of 17.03 ± 5.52 to 14.87 ± 6.24 (*t* = −4.75; *p* < 0.001). A linear regression, conducted separately for the two subgroups of students, showed that the relationship between MA level and mathematics performance was significant for the 31 students whose performance did not improve with time [*F*(29,1) = 19.8; *r* = −0.637; *R*^2^ = 0.406; p < 0.001] but not for the 42 students whose performance improved [*F*(40,1) = 1.564; *r* = 0.194; *R*^2^ = 0.038, *p* = 0.22]. For the students who received no advantage from a repetition of the test, the model accounted for 40% of the variance (Figure 1).

## Discussion

In the present study, the development of acquisition of math skills in a group of 73 students in the first grade of secondary school was followed. For this purpose, a math skills assessment test (the Battery for the assessment of calculation ability ABCA 14-16) was administered for the first time at the beginning and repeated at the end of the year. This battery of math tests is commonly used in Italian schools to identify the specific difficulties encountered by the student in solving mathematical problems as well as to follow the evolution of mathematical skills in relation to age and education. The test score is considered a good measure of the degree of mathematical proficiency.

The investigation aimed to examine whether students’ math anxiety can affect their acquisition of new mathematical abilities. Results highlighted that the score on the anxiety scale, administered at the beginning of the year, negatively correlated with the score obtained on the mathematics test, administered at the end of the school year: the higher the level of anxiety, the worse the performance.

In recent years, special attention has accrued to the possible role played by emotional factors and several studies have documented that MA negatively affects math performance. First, MA induces avoidance behavior towards mathematics, thus consequently reducing learning opportunities. Second, it is documented that MA can interfere at a cognitive function level with both working memory and inhibitory processes (Hopko et al., 2002; Caviola et al., 2012; Mammarella et al., 2018; Skagerlund et al., 2019; Soltanlou et al., 2019; Pelegrina et al., 2020; Van den Bussche et al., 2020; Dowker and Sheridan, 2022). Moreover, studies in which MA has purposefully increased yield poor performance (Kellogg et al., 1999; Galdi et al., 2013), while those in which MA is decreased have improved performance (Furner and Duffy, 2002; Park et al., 2014). However, it should be noted that these data are mainly derived from cross-sectional studies, while the results of studies that have used a longitudinal design are not completely consistent. In our investigation, the level of perceived MA at the beginning of the school year correlated with the score obtained in the end-of-year test, showing that it has a negative effect over time. Interestingly, this negative effect seems to be exerted exclusively on those students who are unable to improve their performance, despite the teaching received and the time elapsed between the two administrations of the same test. Indeed, in the students who did not gain from a retest, the performance had a negative relationship to the MA level. Instead, this relationship was not present in students who improved their skills over time. Given that the improvement in the score obtained on the test is attributable to the achievement of greater competence, the finding of a deterioration in performance, when the task is presented for the second time, can be interpreted as the consequence of an inability to learn how to solve the problems proposed by the math test. Put differently, MA correlated negatively with performance only in students who failed to acquire new knowledge in mathematics over the course of a school year. Ultimately, MA seems to act as a factor that can interfere with the acquisition of new knowledge and skills in mathematics. Furthermore, data allow suggesting the possibility of using an early MA-level assessment to identify students at high risk of poor acquisition of new calculus skills and, consequently, of experiencing difficulties in studying mathematics.

Clearly, our data do not imply that anxiety is the only factor involved in negatively affecting math performance. Certainly, the role of teachers and the family context cannot be overlooked; for example, it has been suggested that MA does not stem directly from the study of mathematics but occurs mainly due to the way mathematics is taught and presented (Turner et al., 2002; Geist, 2010). Moreover, the role of cultural stereotypes should not be underestimated. Cultural stereotypes, which are sometimes transmitted at the family level as well as at the school level, include the idea that if one is not born with a “math gene,” it is useless to strive to study math (Rattan et al., 2012; Goetz et al., 2013; Vukovic et al., 2013). In this regard, it seems fair to emphasize that the characteristics of our sample ensured an acceptable uniformity of contextual aspects: math teachers as well as the environmental context remained unchanged throughout the school year; therefore, these factors should not have significantly affected the results.

Another factor called into question was the age at which the math test was administered. According to some surveys (Wu et al., 2012; Hill et al., 2016; Carey et al., 2019), anxiety increases with age, and the mechanism linking anxiety and performance might be different at different educational stages. In most of the studies, primary and middle school students were examined (Ma and Xu, 2004; Vukovic et al., 2013; Cargnelutti et al., 2017; Gunderson et al., 2018; Geary et al., 2019), while studies on secondary schools were few (Passolunghi et al., 2016; Wang et al., 2020). This variable could justify some discrepancies found in literature surveys (Pekrun et al., 2017; Szczygiel, 2020). From this point of view, it may be helpful to note that the participants in our study belong to the same age group; thus, also the age-related factor does not seem able to influence the results. Furthermore, it should be emphasized that the age of the students on which our study has been conducted, i.e., adolescence, may represent a critical period, both emotionally and behaviorally. With respect to learning mathematics, the first year of secondary school constitutes a time of transition and a turning point between previous experiences and calls for planning an approach to further learning. In this light, conducting the present investigation at this stage of the school journey could be of particular importance in identifying the risk of math difficulties.

Another aspect to consider in interpreting the results is the practice effect, namely, the improvement in performance that is observed whenever the same test is administered several times to a subject; however, the finding that a high percentage of students showed no improvement in performance in the second assessment suggests that this effect cannot account for the data obtained.

In conclusion, our data, in line with what has been described in the literature (Hembree, 1990; Ma, 1999; Maloney et al., 2011; Wu et al., 2012; Núñez-Peña and Suárez-Pellicioni, 2014), confirms the high frequency in which MA occur among students; furthermore, our findings show that MA negatively correlates with the score on the end-of-year math test and suggest that it unfavorably affects the ability to acquire new calculus skills.

Some limitations of this study must be kept in mind. First, the surveyed population consists of students who all attend a technical vocational school where the teaching of mathematics takes on a different value than it does in other educational institutions. Therefore, the results obtained cannot be generalized to other types of students. Indeed, in Italy, mathematics teaching differs quantitatively and qualitatively according to the type of secondary school; the main division is between schools oriented towards the study of humanities, arts, and social sciences and schools more oriented towards physical sciences and technology; in particular, the technical vocational education is oriented towards practical subjects and enable the students to start searching for a job as soon as they completed their studies. It has been proved that Italian students who had chosen science and technology courses show significantly less math anxiety than students who had chosen humanities and social sciences courses (Primi et al., 2014) but little is known about the possible different effects of MA on the math performance of students following different types of study programs (Schommer-Aikins et al., 2015; Barroso et al., 2021). Future studies would be needed to evaluate the extent to which the data obtained are generalizable to various types of courses (Morsanyi et al., 2017; Paechter et al., 2017).

Second, neither the presence of anxiety in the study of school subjects other than mathematics nor the possibility of MA having a negative effect on other study subjects were investigated. It should also be noted that the score of the test was limited to the number of correct answers, according to the instruction manual; other possible measures, such as the time taken to complete the test, have not been considered; the task was carried out during the time for the mathematics lesson as if it were a usual class assignment. Moreover, with regard to the possible mechanisms by which MA is believed to interfere with performance, the present investigation did not include the administration of cognitive tests, such as those related to executive, verbal, and visuospatial functions; however, given that the test administered for the assessment of attainment in mathematics requires adequate working memory functioning (Lucangeli, 1999), the data obtained seem to be in line with what is already known in the literature about the negative influence MA can exert on executive functions (Ashcraft and Kirk, 2001; Hopko et al., 2002; Ashcraft and Krause, 2007; Caviola et al., 2012; Passolunghi et al., 2016; Mammarella et al., 2018; Skagerlund et al., 2019; Soltanlou et al., 2019; Pelegrina et al., 2020; Van den Bussche et al., 2020). Lastly, based on the experimental design used, no conclusion can be inferred about the role played by the difficulty of solving the math test in the genesis of MA, which is a topic that would have required a different experimental design. Similarly, the causal order remains unresolved, that is, whether it is MA that initially reduces performance or *vice-versa* (Ma and Xu, 2004; Carey et al., 2016). It is likely, as suggested by the so-called “reciprocal theory,” that MA and performance continuously exchange roles of cause and effect, thus triggering a vicious cycle and progressively reinforcing each other (Maloney and Beilock, 2012; Carey et al., 2016; Gunderson et al., 2018). In any case, our data suggest that once triggered, regardless of what the “primum movens” was, MA contributes significantly to hindering the acquisition of mathematical skills.

## Conclusion

The growing interest in MA appears to be justified by the fact that acquiring competence in this field takes on value in choosing not only the path of continuation of one’s schooling but also one’s professional future (Dowker et al., 2016; Morsanyi et al., 2017; Carey et al., 2019; Vargas, 2021). A negative attitude to mathematics, in addition to causing a great limitation in life choices (it may lead one to avoid professions that require a commitment to a context related to aspects of mathematics), certainly also entails great difficulties in daily life, especially in social contexts that are highly characterized by technology, such as those of the present day (Suri et al., 2013). Thus, the alarm expressed by international institutions about the decline of math competence in schools seems justified (Invalsiopen, 2022; Save the Children, 2022), especially because the decline is accompanied by an apparent lack of interest in finding solutions (Fondazione Rocca, 2022).

The present investigation can contribute to the understanding that the obstacle to acquiring ever-better math skills may be represented not only by cognitive issues but also, and especially, by emotional issues (Devine et al., 2018; Abín et al., 2020; Passolunghi et al., 2020). Considering anxiety as one of the variables at play in the genesis of learning difficulties may induce teachers to significantly modify their teaching methodology and strategies (Phelps-Gregory et al., 2020), on the one hand abandoning the idea that either you have skills or you do not, and on the other hand gaining awareness that promoting logical reasoning alone is not enough to achieve success (Brown et al., 2008; Devine et al., 2018). There are numerous studies in the literature that aim to propose modes of an intervention designed to take into account the impact of the emotional dimension on learning (Jamieson et al., 2010; Yeager and Dweck, 2012; Brunyé et al., 2013; Park et al., 2014; Supekar et al., 2015; Sokolowski and Necka, 2016; Barroso et al., 2021; Hausman et al., 2021; Samuel and Warner, 2021). From this perspective, an assessment of MA performed during high school entry could become a useful tool to identify which and how many students in a class are at risk of poor acquisition of math skills in the usual course of education. Further studies are needed to confirm this hypothesis, generalize the results, and attempt to answer the many outstanding questions about the relationships between MA and mathematics performance.

## Data availability statement

The raw data supporting the conclusions of this article will be made available by the authors, without undue reservation.

## Ethics statement

The studies involving human participants were reviewed and approved by CEAS Umbria—Comitato Etico Aziende Sanitarie Umbria. Written informed consent to participate in this study was provided by the participants’ legal guardian/next of kin.

## Author contributions

MP contributed to the conception and design of the study and wrote the first draft of the manuscript. GL contributed to the conception and design of the study. CA and SI collected and organized the database. LB, BC, and AL performed the statistical analysis. PD’A and SE contributed to manuscript revision. All authors contributed to the article and approved the submitted version.

## Conflict of interest

The authors declare that the research was conducted in the absence of any commercial or financial relationships that could be construed as a potential conflict of interest.

## Publisher’s note

All claims expressed in this article are solely those of the authors and do not necessarily represent those of their affiliated organizations, or those of the publisher, the editors and the reviewers. Any product that may be evaluated in this article, or claim that may be made by its manufacturer, is not guaranteed or endorsed by the publisher.
